# Sexual and reproductive health challenges among adolescents and young people with spina bifida and hydrocephalus disability in Uganda: A qualitative study

**DOI:** 10.1371/journal.pone.0308194

**Published:** 2025-05-27

**Authors:** Denis Ndekezi, Andrew Sentoogo Ssemata, Ambrose Ganshanga, Ruth Nalugya

**Affiliations:** 1 Non-communicable Diseases Theme, MRC/ UVRI & LSHTM Uganda Research Unit, Entebbe, Uganda; 2 Spina Bifida and Hydrocephalus Association of Uganda (SHAU), Kampala, Uganda; 3 Department of Global Health and Development, London School of Hygiene and Tropical Medicine, London, United Kingdom; Indiana University School of Medicine, UNITED STATES OF AMERICA

## Abstract

**Introduction:**

Globally, 180 million young people aged 10–24 live with a physical or mental health disability. Their rights to sexual and reproductive health have been denied often overshadowed by the societal negative knowledge, beliefs, and attitudes. This study sough to explore sexual and reproductive health challenges among adolescents and young people with spina bifida and hydrocephalus disability in Uganda.

**Methods:**

This was an exploratory community based cross-sectional qualitative study among adolescents in Uganda. We conducted 60 semi structured interviews, 30 with Adolescents and young people with spina bifida and hydrocephalus and 30 with caregivers between March 2021 and February 2022 to explore their sexual and reproductive health challenges. Through thematic analysis, we identified common themes across the interviews regarding their challenges.

**Results:**

Participants reported a number of sexual and reproductive health challenges including sexual violence and abuse, incontinence and stigma, Inability to feel sexually empowered, other adolescents are coerced to take birth control methods without their informed decision. Negative Socio-cultural beliefs, lack of and inaccessible sexual reproductive health information and poor menstrual health management.

**Conclusion:**

Adolescents and young people with spina bifida experience a number of sexual and reproductive challenges that are not given attention and often overshadowed by the negative knowledge, beliefs, attitudes, and practices within the society. Therefore, there is need to develop and implement programs and awareness campaigns aimed for the empowerment of individuals with SB to seek knowledge and skill building regarding sexual and reproductive health.

## Introduction

Globally, 16 percent of the global population is persons with disabilities [1] and almost 180 million young people aged 10–24 live with a physical or mental health disability [2]. A significant proportion, 80%, of these young individuals are based in countries characterized by low incomes and limited financial resources [3]. Research has revealed that persons with disabilities have historically been denied their sexual and reproductive health (SRH) rights [4]. Some of these include less access to SRH information, which is necessary for healthy and safe relationships, protection from HIV and other STIs, and realization of sovereignty in family planning decisions [4,5]. A descriptive review to assess the effectiveness of initiatives to improve adolescent access to and utilization of sexual and reproductive health services in low- and middle-income countries revealed that the different forms of discrimination and challenges that women with disabilities often experience, many of which increase their vulnerability to different forms of violence [6]. lack of confidence in their abilities to express their sexual needs, fears of rejection [7] and worry about incontinence [8] and failure to manage sexual relationships contribute to poor health outcome and inability to participate fully in their daily growth activities. Additionally, adolescents and young people (AYP) in Uganda face challenges related to sexual rights, abuse, and socio-cultural practices. A study revealed that cultural beliefs often associate disabilities with witchcraft or religious punishment, leading to stigma and discrimination that negatively impact the sexual rights and well-being of individuals with spina bifida [9]. For adolescents and young people with spina bifida and hydrocephalus (SBH), a congenital conditions that impact physical and neurological development, their life presents with complications including urinary tract infections, pressure sores, anaemia, physical and sexual abuse risks and developmental disabilities [10]. Despite these challenges, their health needs often receive inadequate attention and are overshadowed by widespread negative knowledge, beliefs, attitudes, and practices [11]. A systematic review of 78 articles revealed significant regional and socioeconomic disparities in the incidence of congenital hydrocephalus, with the highest rates observed in Africa and Latin America, at 145 and 316 per 100,000 births, respectively, and the lowest rate in the United States, Canada, at 68 per 100,000 births [12].

In Uganda, spina bifida and hydrocephalus remain significant public health concerns. A study analyzing 110,752 births in the country reported a spina bifida prevalence of 5.7 per 10,000 births, making it the most common neural tube defect in the cohort [13]. Additionally, research conducted in southwestern Uganda found that 87% of the 235 patients with neural tube defects had spina bifida, further emphasizing its burden in the region [14] However, there is a lack of sex-specific data on its occurrence. Studies indicate that individuals with hydrocephalus are less likely to engage in sexual activity and experience normal sexual function compared to those without the condition [15]. In Uganda, a study assessing the quality of life among children with spina bifida and hydrocephalus found that factors such as sex, hydrocephalus, mobility, and continence status significantly influenced their quality of life [16]. While this study did not specifically address sexual health, it highlights the importance of considering these factors in the overall well-being of individuals with spina bifida and hydrocephalus. The high prevalence of spina bifida and hydrocephalus in Uganda, coupled with the societal stigma and misconceptions surrounding these conditions, exacerbates the challenges faced by affected individuals, particularly in accessing comprehensive sexual and reproductive health education and services. This highlights the urgent need for targeted interventions and awareness campaigns at the local level to promote inclusivity, address stigma, and ensure the sexual and reproductive rights of individuals with spina bifida and hydrocephalus are respected and protected. However, exploration of their SRH challenges under such circumstances remains low especially in low and middle-income countries. Currently, most research on spina bifida and hydrocephalus focuses on the sexual health and sexual satisfaction among adults [7,17,18] and little is known about the SRH challenges among the adolescents and young people with Spina bifida and hydrocephalus. Recognizing the distinct obstacles, vulnerabilities, and strengths of adolescents and young people with SBH can help ensure that relevant authorities effectively address and protect their sexual and reproductive health rights. The aim of this study was to explore sexual and reproductive health challenges among adolescents and young people with spina bifida and hydrocephalus disability in Uganda.

## Materials and methods

This was an exploratory community based cross-sectional qualitative study among adolescents, young people living with spina bifida and hydrocephalus and their caregivers (parents and siblings). This study took place in the central region urban (Kampala & Wakiso) and Peri-urban and Rural (Mukono & Mityana) areas of Uganda. The central region of Uganda was selected for its diverse urban, peri-urban, and rural settings, allowing for a representative sample. Kampala and Wakiso represented urban areas, while Mukono and Mityana represented peri-urban and rural areas. This geographic diversity enabled exploration of variations in experiences, access to healthcare, social support, and resources across different settings, providing a comprehensive understanding of challenges and opportunities faced by adolescents and young people living with spina bifida and hydrocephalus in Uganda.

### Sampling and research participants

Working with Spina bifida and Hydrocephalus Association of Uganda (SHAU), a national coordinating body, we purposively identified and selected young people, adolescents and caregivers (Parent/ caregiver or a sibling - those involved in the care and management of children with spina bifida and hydrocephalus) from the association registers who belong to the Central region. The association supported the first contact with potential participants, and these participants were contacted and information about the study shared with them. Participants were informed about the study procedures and made to understand that participation is voluntary and refusal to participate would not attract any penalty or adversely impact their relationship with the Association. One participant declined to participate but all the other participants contacted accepted the invitation and were scheduled for consenting and interviews according to their date, time, and location preference. All participants in the study had a possibility of movement despite the condition and none of the participant was immobilized in bed. This qualified all participants to be eligible for the study.

### Ethical considerations

The study obtained ethical approval from the Mildmay Uganda Research Ethics Committee (REC Ref 0710–2020) and the Uganda National Council for Science and Technology (SS703ES).

Before conducting the interviews, participants who were 18 years or older provided written informed consent, while those under 18 gave written informed assent and had their parents or guardians provide consent. Additionally, all participants gave consent for audio recording. To maintain confidentiality, children were interviewed in a private space away from the caregivers and family members. Participants were assigned pseudonyms, and any identifiable information was excluded from the transcripts. Children who had difficulties in reading, were given easy to ready study documents. The researchers provided comfort breaks for participants during data collection to refresh, manage fatigue and sensory overload. This was to ensure that participants remained comfortable, engaged, and able to contribute meaningfully to the study.

### Data collection

Qualitative in-depth interviews (IDIs) were conducted face to-face by an experienced researcher to guide the data collection process as a formative evaluation means to explore the sexual and reproductive health challenges among adolescents and young people living with SBH. Participants were recruited through purposive sampling with the help of the association co-ordinator between March 2021 and February 2022. The IDIs were held in a conducive place that was safe, neutral and with minimal distractions for the participants and the researcher. This place was either suggested by the interviewee or preset by the interviewer in the community.

We conducted 30 IDIs with the adolescents and young people living with SBH and 30 IDIs with the Caregivers (parents, siblings involved in taking care of the AYP living with SBH) in the central region urban (Kampala & Wakiso) and Peri-urban and Rural (Mukono & Mityana) areas of Uganda using semi-structured interview guides (S1 File and S2 File) to obtain in-depth descriptions insights about their SRH challenges.

Data collection was conducted in English and/or Luganda (a commonly used dialect in Central Uganda). All the IDIs, conducted by the first two authors (DN, AS) were audio-recorded using a digital recorder and averagely lasted 60 minutes. The recordings were transcribed verbatim and those in Luganda were translated to English and reviewed by the third author (RN) to ensure accurate translation and transcription to avoid losing original stories.

We employed a saturation-based approach to determine the sample size of 60 participants (30 caregivers and 30 adolescents and young people with spina bifida) for the interviews. We initially aimed to purposively interview at least sixty participants and continued data collection until no new themes emerged. Forty participants (20 caregivers and 20 adolescents with Spina bifida) were interviewed, and thematic saturation was achieved. Twenty additional interviews (10 caregivers and 10 adolescents with spina bifida and hydrocephalus) were conducted to confirm the themes and insights obtained from the initial interviews.

### Data management and analysis

In-depth interviews were audio recorded, transcribed verbatim, and then translated into English 

for a hybrid approach of inductive and deductive thematic content analysis [19] by two researchers (DN and AS) experienced in qualitative methodology and disability research. 

Transcripts were read through for familiarization and coded manually by DN and AS. To ensure coding consistency, the developed codes were shared with the study principal investigator (RN) to facilitate collaborative thematic analyses throughout [20]. Transcripts were then imported to NVIVO 12 software to assist in coding and identifying themes.

Themes and sub-themes were continually reviewed and refined to capture emerging new codes. Quotes were captured to highlight thematic areas and increase our understanding of the context.

## Results

The study involved 60 Participants, 30 caregivers of children with spina bifida and hydrocephalus, with caregivers’ ages ranging from 15 to 61 years and diverse education levels and occupations. The children’s ages ranged from 11 to 23 years, with most having lower primary education and a balanced gender distribution as shown in table 1.

**Table1:** Showing the participant demographic characteristics.

Participant ID	Caregiver-Category	Age in years	Education level	Occupation	Gender of child with SBH	Age of child with SBH	Education level of the child with SBH
01	Mother	54	Primary	Unemployed	Male	14	Lower Primary
02	Mother	36	University	Unemployed	Male	16	Lower Primary
03	Grandmother	61	University	Retired	Male	23	Upper Secondary
04	Mother	53	Primary	Hand Crafts vendor	Female	15	Lower Primary
05	Father	30	University	Skills Trainer	Female	11	Lower Primary
06	Mother	57	No education	Market Vendor	Male	23	Lower Primary
07	Mother	42	Lower Secondary	Casual worker	Female	16	Lower Primary
08	Mother	43	Primary	Unemployed	Female	14	Lower Primary
09	Father	27	University	Farmer	Male	20	Lower Primary
10	Sibling	15	Primary	Home Caretaker	Male	13	No education
11	Sibling	16	Lower secondary	Casual worker	Male	14	Lower Primary
12	Mother	40	Lower Secondary	Farmer	Male	16	Lower Secondary
13	Sibling	15	Lower Secondary	Student	Male	21	Lower Primary
14	Sibling	33	University	Social worker	Female	18	Lower Primary
15	Sibling	15	Lower Secondary	Student	Female	13	No education
16	Mother	28	University	Teacher	Female	11	Lower Primary
17	Mother	54	No education	Market Vendor	Male	23	Lower Primary
18	Mother	37	Lower Secondary	Casual worker	Female	16	Lower Primary
19	Father	36	Lower Secondary	Unemployed	Female	12	Lower Primary
20	Mother	30	Primary	Food vendor	Female	14	Lower Primary
21	Mother	43	Lower Secondary	Unemployed	Female	15	No education
22	Father	38	University	Physical Planner	Male	13	Lower Primary
23	Mother	30	Primary	Unemployed	Male	12	No education
24	Mother	27	University	Salon attendant	Female	14	Lower Primary
25	Sibling	18	Upper Secondary	Student	Male	15	Lower Secondary
26	Sibling	23	University	Student	Male	17	Upper Secondary
27	Sibling	16	Lower Secondary	Student	Male	18	Lower Secondary
28	Sibling	15	Lower Secondary	Student	Female	13	No education
29	Mother	28	University	Salon attendant	Female	11	Lower primary
30	Father	54	No education	Market Vendor	Male	23	Upper Secondary

During data analysis we identified seven themes and their corresponding excerpts that highlight the sexual and reproductive health challenges among the adolescents and young people with spina bifida and hydrocephalus. Sexual violence and abuse in the society, incontinence and stigma, inability to feel sexually empowered, coerced birth control uptake, negative Socio-cultural beliefs and practices, lack of or inaccessible SRH information, services and poor menstrual health management were identified as the major SRH challenges people with spina bifida and hydrocephalus face in Uganda.

### Sexual violence and abuse in the society

Adolescents and young people living with SBH reported experiencing some form of sexual violence and abuse from the community members these children attributed these provocations to their disability, as the perpetrator often referred to the child’s disability when carrying out the maltreatment. For example, one of the participants stated:

*“The vulnerability I see is more about us girls with this condition [spina bifida and hydrocephalus, because there is a disabled girl who is one of my friends that was raped because of her inability to protect herself and she has spina bifida.”* Female, person with disability.

In some cases, these perceptions of the community that people living with spina bifida and hydrocephalus do not deserve to live has exacerbated the violence as one of the caregivers narrate the agony her child face in the society.

*“You see one of the risks or the vulnerabilities they have is that some members in the society look at them as people who are not important in the society. You have heard of stories of them being raped, sexually abused because they know they cannot fight back.”* Female caregiver

Some participants believed that the differences in the treatment of children with disabilities could be attributed to the greater participation of children with physical impairments in society, such as going to school and participation in daily income activities.

### Incontinence and stigma

Young people with spina bifida and hydrocephalus reported experienced incontinence as challenge to their sexual health that affects their self-worth, emotional well-being, and their overall quality of sexual life. The leakage exposes them to many physiological problems such as embarrassment, guilt, anxiety, fear about sex and this makes many of them to avoid getting in relationships.

*“For me I feel scared to have a boyfriend… I have spina bifida and one of the challenges is that I cannot control my urine flow. Because of this [incontinence] I feel it is normal not to have a boyfriend.”* Female, Person with disability

*“One thing is that, if you want to have sexual intercourse and your partner picks that smell of the urine, she will just leave you.”* Male, Person with disability

Some participants in romantic relationships faced stigma and bullying from their partners’ families, who disapproved of their relationship due to the participant’s disability. The in-laws viewed the participant as a burden and did not want their child to be “encumbered” with someone with a disability, as one participant shared.

*“His family bullied me, and I went through depression for like two months. They were like, how can you bring someone with a disability [spina bifida and hydrocephalus], she is going to be a burden in all your life.”* Female, Person with disability

*“…once the person gets to know that you have a disease [spina bifida or hydrocephalus], they will just run away and they will never return.”* Female, Person with disability

Overwhelmed by the circumstances and the stigma, some caregivers also noted that stigma grossly affects the children’s esteem and develop negative self-concept that influence their life, for example self-isolation, fear to engage in health relationships and resort to other mechanisms like masturbation for sexual pleasure.

*“I told him that he will get sick if he continues doing that [masturbating], but he refused. I even do not know if he will produce children when he gets a wife because according to what people say, masturbation kills the potential of someone to produce children, yet he has done it for over 20 years. He tells me he masturbates because he fears to approach women for sexual relationships.”* Female, caregiver

### Inability to feel sexually empowered

Male participants were shy to discuss their sexual functionality especially when asked if they always encounter penile rigidity. However, while interacting with the caregivers and the siblings of the people living with spina bifida and hydrocephalus, most them were concerned about the sexual life of their children and they noted that most of them have never experienced an erection just like any other normal male child and this is the major sexual challenges they are experiencing.

*“He is becoming a teenager now and he does not have a penile erection like other boys and this might be a challenge in his sex life.”* Female caregiver.

*“…most times I see his private part in between his thighs and even when he is urinating, he sits and pushes himself to urinate. I have never seen him erecting like a normal man and that is the only challenge I see.”* Male Sibling

*“I share a room with him, but I have never seen him [child with spina bifida and hydrocephalus] when he has reacted [with a penile erection] like men, you understand.”* Male Sibling.

Some of the caregivers reported male fathers do not spare time and talk about all these sexual issues with their children and mothers believe this affects these children. They added that male and female children with SBH need the sexual and puberty education like the other normal children to understand their body changes or any other challenge they face. These concerns highlight the need for open and honest communication between caregivers, particularly fathers, and children with spina bifida and hydrocephalus about sexual health and development. Providing puberty education and support can help alleviate concerns and empower individuals with SBH to understand their bodies and make informed decisions about their sexual health.

### Coerced birth control uptake

Forced birth control uptake was another major finding reported by some of the female participants during the interviews. Most of them due to their life challenges and experiences they are forced by their caregivers to take birth control methods without an informed decision and this is meant to stop them from having children especially as one of the participants narrates in the excerpts below.

*“They forced me to get a family planning injection for birth control so that I do not give birth to a child who will be a burden to my parents like me.”* Female, Person with disability

While other parents hope and pray for their children to have successful families of their own, caregivers of children living with spina bifida and hydrocephalus wish for the opposite- they hope their children do not give birth to children as this will be an added responsibility onto them that would further burden both the child and themselves. Caregivers perceive that these children are entirely dependent, and they cannot take care of themselves, and let alone raise a child if they gave birth.

*“I would not want my child to be sexually active or to give birth… Because it will be like I am the one who has given birth again. You see, she cannot grip; she cannot do anything on her own. I am the one who does everything for her, so if a man does something like that [makes her pregnant], it is like he is damaging my life.”* Female -caregiver

### Negative Socio-cultural beliefs and practices

Adolescents and young individuals with spina bifida and hydrocephalus face significant obstacles due to harmful cultural attitudes, which often prevent them from participating in the workforce and make them vulnerable to sexual exploitation.

*“I went for a job interview and my boss told me that, in his culture, when you sleep with someone with a disability, all the bad luck will go away. I was like, if you do not want to give me your job, I will leave because after that [sexual encounter], what will my dignity be like.”* Female -person with disability.

When it comes to maternal and childbearing stages some of them are told false beliefs that they cannot conceive and this exposes them to sexual activities with men to prove if they cannot conceive as some of the caregiver explains in the excerpts below.

*One child with spina bifida and hydrocephalus told me that people were saying that she will not be able to conceive and give birth to a child, this forced her to have sexual intercourse with a man who was far older than her so as to prove and see if what people were saying was true.* Female -caregiver

*“People think that since we have spina bifida and hydrocephalus, we are mentally ill, and we do not have any sexual and reproductive health needs at all. They do not even call us for the youth meeting or even for community outreach services.” Male -Person with disability.*

### Lack of or inaccessible SRH information and services

Both male and female respondents noted inherent societal expectations and mis-perception that people with spina bifida and hydrocephalus do not need SRH services including SRH information. Consequently, there is over-protection of children with physical disabilities especially girls through denial of information as the following quotation illustrates:

*“In relation to reproductive health, I have not received any help from the health workers or even the family members. Because even our parents do not allow us to go to hospitals”* Male -person with disability.

Lack of information is in part influenced by the caregiver and the sibling they stay with at homes. Some deliberately deny them the right to accessing the information or to take them to places where they can access the information and the services as some of the participants narrates in the excerpts below.

*“He does not know those things [sex related issues] or services related to SRH because it is you to take him to the hospital.”* Female -sibling

*“She might be having little knowledge related to puberty and reproductive health. The only time I have taken her to hospital is for a blood test after that instance of the boy trying to rape her. Since then, I have never taken her back, for things concerning HIV testing or protection against STDs. She does not know them at all.”* Female -caregiver

Additionally, the access to sexual and reproductive information was affected by the parent’s limited knowledge about these services, and the widespread belief that SRH services are not suitable for people with spina bifida and hydrocephalus due to their short life expectancy as one of the care-givers stated.

*“Some parents lack knowledge and feel it is not necessary for their children [with spina bifida or hydrocephalus] to participate in sexual and reproductive health services because they might not live for long due to their health condition.”* Female -caregiver

As a result, young people who are pregnant face challenges in obtaining antenatal and childbirth care services. Participants in the study highlighted the limited availability of healthcare facilities, noting the absence of specialized care services for antenatal and postnatal periods, as well as the lack of welcoming facilities, such as adequate labour beds in health units.

*“Another thing is even when we get pregnant, our health facilities are not suitable for us. For example, the beds in the labour ward are not suitable for mothers with spina bifida or even hydrocephalus [un-adjustable beds], they are meant for normal people which also becomes a challenge for us to access such services.”* Female -person with disability

*“I cannot access specialized services. For example, when I go to the hospital, I am tired of seeing these general medical doctors. One day they did a scan, and I asked a doctor, ‘are my ovaries ok? Am I able to carry a child?’ He was like what!!! Do you mean you can carry a child? He was amused.”* Female -person with disability.

### Poor menstrual health management

Adolescent girls and young women living with spina bifida and hydrocephalus issues expressed strong negative emotions towards their menstrual experiences, describing feelings of discomfort, anxiety, and self-consciousness about bleeding accidents while socializing with friends in school. They also reported inadequate social support and lack of access to proper menstrual products, leading some to resort to using unsanitary materials, as shared by one participant.

*“I regretted having periods at an early age, I did not have enough information and right menstrual materials to use. I resorted to using newspapers because I feared to ask for support from my friends.”* Female, person with disability

*“She lacks the right menstrual materials to use and there are other girls like her that lack materials, which worries them. They end up using unhygienic materials.”* Female - caretaker.

The negative emotions of “hate” and “fear” associated with menstruation can have far-reaching consequences for girls’ self-assurance, self-worth, and social interactions during puberty. Furthermore, the erosion of self-esteem during menstruation can have a ripple effect on other reproductive health issues, as girls who lack confidence may struggle to express their needs and concerns, fearing judgment and stigma from their environment, leaving them marginalized and unsupported.

## Discussion

This study highlights the sexual and reproductive health challenges faced by adolescents and young people living with spina bifida and hydrocephalus in Uganda. These encounter including sexual violence, incontinence, stigma, coerced contraception, and poor menstrual health management. These issues are compounded by societal misconceptions, cultural beliefs, and systemic neglect, creating an environment where individuals with spina bifida and hydrocephalus are marginalized and denied their fundamental SRH rights. Addressing these challenges requires a dual approach: empowering individuals with spina bifida and hydrocephalus to advocate for their rights and ensuring that society, including policymakers and healthcare providers, fosters an inclusive and supportive environment.

Adolescents and young people living with spina bifida and hydrocephalus in this study reported experiencing sexual violence and abuse, from the community members and the participants attributed these provocations to their disability, as the perpetrator often referred to the child’s disability when conducting the maltreatment. which highlight that women with spina bifida are particularly vulnerable to sexual violence and exploitation [8]. The study further reveals that sexual violence against individuals with spina bifida and hydrocephalus remains a silent epidemic, often ignored within reproductive health discussions and broader policy frameworks. Some participants reported that these experiences increased their vulnerability to secondary disabilities, such as trauma-induced mental illness [21] possibly reflecting internalised stigma, low self-confidence and desire for social and sexual acceptance [18]. These findings underscore the urgent need for targeted sexual abuse prevention strategies and education programs tailored to the needs of adolescents and young people with spina bifida and hydrocephalus.

Urinary and faecal incontinence emerged as significant barriers to the sexual health and self-esteem of adolescents and young people with spina bifida and hydrocephalus. Participants described incontinence as a major source of embarrassment, guilt, and anxiety, negatively impacting their emotional well-being and sexual relationships [7,8]. Previous studies have similarly reported that individuals with incontinence are less likely to engage in sexual activities due to fear of rejection and feelings of inadequacy [22]. The psychological burden associated with incontinence emphasizes the importance of targeted interventions, including access to appropriate medical management, psychological support, and open discussions on sexuality for individuals with spina bifida and hydrocephalus.

Negative cultural beliefs and perceptions significantly impact the SRH rights of adolescents and young people with spina bifida and hydrocephalus. Many participants reported societal beliefs that individuals with spina bifida and hydrocephalus cannot conceive due to the impact of SBH on their sexual function Negative cultural perceptions significantly impact the SRH rights of adolescents and young people with spina bifida and hydrocephalus. Many participants reported societal beliefs that individuals with spina bifida and hydrocephalus cannot conceive due to their condition [23] or that they bring good luck to men after sexual intercourse, leading to sexual exploitation. The negative cultural belief contribute to the dehumanization of individuals with SBH and expose them to increased sexual abuse. Addressing these issues requires efforts to educate both individuals with SBH and the broader community on SRH rights while building confidence among adolescents and young people with SBH to demand implementation of appropriate policies that protect their dignity and autonomy.

A concerning finding was the forced administration of contraceptive methods to adolescents and young people with spina bifida and hydrocephalus without their informed consent, reflecting a broader violation of their reproductive rights. Similar trends have been reported globally, where individuals with disabilities are often subjected to forced sterilization, coerced contraception, and limited access to reproductive healthcare [3]. This study highlights how people with spina bifida and hydrocephalus are systematically excluded from making decisions about their reproductive health, facing barriers such as poorly managed pregnancies, limited access to maternal care, and involuntary abortions. Addressing these violations requires an urgent review of policies to ensure that family planning services are inclusive, consent-driven, and respectful of the rights of individuals with disabilities.

Menstrual health management was another significant challenge faced by adolescents and young people with SBH, particularly due to physical limitations, lack of appropriate menstrual products, and limited social support. Participants reported resorting to unhygienic alternatives such as cloth, cotton, or toilet paper, exposing them to infections and further discomfort. These findings align with global studies highlighting disparities in menstrual health management among individuals with disabilities [24]. The lack of clear information and support systems surrounding menstrual health exacerbates feelings of isolation and helplessness among young women with spina bifida and hydrocephalus. There is a need for further research to develop menstrual products that are comfortable, accessible, and suitable for individuals with disabilities, as well as initiatives that provide clear guidance on product options and safe disposal methods.

### Challenging stigma and promoting disability-inclusive SRH services

A major theme emerging from this study is the role of societal attitudes in reinforcing the SRH challenges faced by adolescents and young people with spina bifida and hydrocephalus. Many participants emphasized that societal misconceptions falsely assume that individuals with spina bifida and hydrocephalus do not require SRH services, leading to their exclusion from comprehensive SRH education and care [3,18]. This marginalization perpetuates poor health outcomes, lack of awareness, and increased vulnerability to sexual exploitation. Addressing these barriers requires a shift in societal perceptions through community education campaigns, disability-inclusive SRH policies, and proactive involvement of healthcare providers in addressing the unique needs of individuals with SBH.

### Improving access to healthcare and policy implementation

Limited access to antenatal and childbirth care services for pregnant young women with SBH was another critical issue identified in the study. Participants reported difficulties in accessing healthcare due to unfriendly facilities, lack of specialized maternal care, and inadequate policy implementation. These findings align with a previous qualitative study conducted in Kampala, which reported similar barriers for individuals with disabilities [17]. The absence of accessible antenatal and postnatal care services contradicts internationally recognized SRH rights [25,26], further entrenching social exclusion. To address this, policies aimed at improving SRH access for individuals with spina bifida and hydrocephalus must be actively implemented and enforced to ensure tangible benefits for this population.

### The need for comprehensive sexuality education and disability-inclusive programs

The study highlights a significant gap in SRH education and information for adolescents and young people with spina bifida and hydrocephalus. The lack of specialized training and resources means that individuals with SBH often struggle to navigate their sexual health and relationships, increasing their risk of negative health outcomes such as sexually transmitted infections (STIs), HIV, unintended pregnancies, and sexual exploitation (24). Integrating disability-specific SRH education into mainstream programs is crucial to equipping individuals with the knowledge and skills necessary to make informed decisions about their sexual and reproductive health.

### Strengths and limitation of the study

This was a qualitative study that involved a small number of participants; therefore, the results may not be empirically generalizable to all the adolescents and young people with SBH in Uganda but rather conceptually generalizable. We recruited and enrolled a limited number of adolescents and young people with spina bifida and hydrocephalus based on the organization registers for central region urban (Kampala & Wakiso) and Peri-urban and Rural (Mukono & Mityana) areas of Uganda thus these may not be representative of all persons with SBH disability in Uganda. It is likely that the challenges faced by adolescents and young people in central region of Uganda might be different from those experienced by adolescents and young people with spina bifida and hydrocephalus living in other regions of the country. We neither used complex theories nor sought to develop theory derived from the data but used robust framework analysis techniques to generate the major themes.

Our study provides critical insights into the sexual and reproductive health challenges faced by adolescents and young people with spina bifida and hydrocephalus in the central region of Uganda. While the findings may not encompass all experiences of adolescents and young people with spina bifida and hydrocephalus, they offer a crucial perspective on the specific difficulties this marginalized group encounters. This research highlights the urgent need for tailored policies and interventions to address these challenges. By shedding light on these challenges, our study advocates for a comprehensive approach to SRH education, services, and support, contributing to the empowerment and well-being of adolescents and young people with SBH. The findings help to highlight the need for improving SRH Environment for adolescents and young people with spina bifida and hydrocephalus as well as changing health the cultural and community attitudes towards them. Additionally, participants were open in talking about their sexual and reproductive health challenges which allowed for the development of novel concepts and enabled the attainment of thematic saturation, a prominent strength of the study.

## Conclusion

This study highlights the significant sexual and reproductive health challenges faced by adolescents and young people with Spina Bifida and Hydrocephalus, including sexual violence, coercion into contraceptive use, incontinence, stigma, and limited access to SRH information and services. These challenges are compounded by harmful socio-cultural beliefs that marginalize individuals with disabilities, restricting their autonomy and well-being. Addressing these issues requires a multi-faceted approach. Raising awareness through community education can help dismantle harmful misconceptions and reduce stigma surrounding the sexuality of individuals with SBH. Policy and legal reforms should prioritize the protection of their SRH rights, ensuring access to comprehensive, disability-inclusive reproductive health services. Additionally, improved menstrual health management strategies must be developed to meet the specific needs of young people with spina bifida and hydrocephalus. Future research should explore context-specific interventions to promote SRH education, enhance social inclusion, and empower individuals with spina bifida and hydrocephalus to make informed choices about their reproductive health. By fostering an inclusive and supportive environment, we can ensure that all individuals with disabilities, including those with spina bifida and hydrocephalus, have the resources and rights they need to lead dignified and empowered lives.

## Supporting information

S1 File Interview guide for participants with disability. (PDF)

S2 File Interview guide for siblings and caregivers. (PDF)
