# Supplementary material for: Sexual and reproductive health challenges among adolescents and young people with spina bifida and hydrocephalus disability in Uganda: A qualitative study
Source: PLoS One. 2025 May 27;20(5):e0308194. doi: 10.1371/journal.pone.0308194 (PMC12111527; doi:10.1371/journal.pone.0308194)
Supplement: S1 File — (PDF) [file pone.0308194.s001.pdf]

SPINA BIFIDA &HYDROCEPHALUS ASSOCIATIONS OF UGANDA
Willis Road Namirembe, 100m off Namirembe Cathedral, Kampala Uganda.
Tel: +256 454 435273/435356

Exploring and understanding the life skills, sexual, reproductive and mental health needs among children and adolescents with disability in Sub Saharan Africa; a mixed methods study (REMHAND Study)

In-depth Interview guide for participants with disability

[EXPLAIN THE FOLLOWING]

- This will be a 45mins-1hr activity that will be audio-recorded.
- I would like to hear your views/story. There are no right or wrong answers.
- Please feel free to give your ideas
- Any question you feel uncomfortable about, please feel free not to answer it. However, for learning, we would like to hear everything you want to say. Therefore, if this is of no harm to you, try and tell us everything.
- Your names will be kept confidential – when we write up the discussion, we never use people's real names. Instead, we will allocate a number which we shall use to identify you.
- The information you share with me will be between the two of us (emphasise that other study staff will not know what participant tells you)
- I will be happy to answer any questions that may come up
- Please put your cell phone/s on silent if possible
- Is it okay with you that we start the interview?

Note: Record the date and time activity starts.

Social Demographics

1. Please tell me a little bit about yourself. (Probe for when the participant was born, education, marital status, occupation, ethnicity, number of children among others.

General Questions

2. Share with me and describe the forms of disability that you are aware of in your community Probe for Physical, mental, do they have any PWDs at their home, experience living with or having PWDs in a home.
3. Please share with us what it means to care or live with for a person with disabilities? (probe for the challenges they go through, how they try to deal with the challenges, and what age is the most challenging for PWDs, and why? what can be done to help people with disabilities live a meaningful lifestyle?)

4. What are some of the needs of PWDs in the families and communities where you live? [Probe for skills development, SRHs, Mental health services] Are these services necessary for PWDs?
5. What are the facilitators and barriers that affect access to the mentioned services above for PWDs? (please ask the facilitators differently and the barriers differently)

Sexual and Reproductive Health

6. Is HIV a risk for you? Have you ever tested for HIV? (Probe for reasons for testing or not testing)
7. Please share with me the insight into the development of your sexuality. [*probe for factors shaping this development including parental attitudes, peer pressure and cultural norms*].
8. How was your experience the first time you had sexual intercourse? [*The context of first intercourse and risk taking behaviour, control over the tempo and activities on that occasion, and, more generally, in their sexual dealing up to that point*].
9. If the respondent has not yet engaged in any sexual activity, what are the reasons why a respondent has yet experienced first intercourse. [*Probe for; How they feel about their current status, what do other people say about it?*]
10. Please recall and describe your sexual history. [*feelings and relationships, since first intercourse, including detailed accounts of more recent events and relationships*.] *Particular attention should be paid to detailed descriptions of recent interactions, where possible, including examples of both safer and less safe activities so that comparisons can be made. The use of contraception is explored in detail, as well as perceptions of risk, vulnerability, and related issues.*
11. What are the risks for you in relation to sexual health?
12. Please share with us your risk taking behaviour, perceptions of risk and vulnerability as well as the mechanisms employed to avoid risk.
13. What is your knowledge, attitudes and usage of the sexual health services provided for young people in yourr locality? [*Including health centre based services and those provided by youth organisations. Opinions are sought on the appropriateness of the services and facilities offered as well as on their personal experiences and ideas on how services could be improved upon.*]

Mental Health

14. What are the factors that encourage good mental health (social, economical, political etc)? *Probe for factors damage mental health and how are these changing over time*.
15. What factors reduce the prevalence of mental health problems or increase the prevalence of good mental health, how can we improve recovery and support for individuals who are unwell.
16. How does mental health affect different groups of people or cause inequalities? *what are the community's main assets to deal with mental health problems and how strong are social networks? what do local people feel about mental health and what are they concerned about? how do these wider factors affect the need for mental health services?* how can these wider influences on health be tackled by prevention initiatives?

COVID-19

17. What do you know about COVID-19?
18. What were your experiences and feelings in seeking care and services during the COVID-19 pandemic and lockdown?",
19. Do you think the COVID-19 pandemic has affected your health and care seeking behaviours ? probe to explain how.
20. How did you cope or are you coping with the difficulties of care and support during the COVID-19 pandemic?
21. What can you do to prevent the COVID-19 disease?
22. Is there anything we might have forgotten to ask that you would like to talk about?

Thank you for sharing these important views about PWDs.
